# Fecal Microbiome and Urine Metabolome Profiling of Type 2 Diabetes

**DOI:** 10.4014/jmb.2411.11071

**Published:** 2025-03-11

**Authors:** Hye-Min Yi, Seok Won, Juhan Pak, Seong-Eun Park, Mi-Ri Kim, Ji-Hyun Kim, Eun-Young Park, Sun-Young Hwang, Mee-Hyun Lee, Hong-Seok Son, Suryang Kwak

**Affiliations:** 1 College of Korean Medicine, Dongshin University, Naju 58245, Republic of Korea; 2 Dangbom Korean Medicine Clinic, Seoul 03192, Republic of Korea; 3 Department of Bio and Fermentation Convergence Technology, College of Science and Technology, Kookmin University, Seoul 02707, Republic of Korea; 4 Department of Biotechnology, College of Life Sciences and Biotechnology, Korea University, Seoul 02841, Republic of Korea

**Keywords:** Type 2 diabetes, fecal microbiome, urine metabolome, biomarker

## Abstract

Type 2 diabetes is a prevalent metabolic disorder with serious health consequences, necessitating both enhanced diagnostic methodologies and comprehensive elucidation of its pathophysiological mechanisms. We compared fecal microbiome and urine metabolome profiles in type 2 diabetes patients versus healthy controls to evaluate their respective diagnostic potential. Using a cohort of 94 subjects (48 diabetics, 46 controls), this study employed 16S rRNA amplicon sequencing for fecal microbiome analysis and GC-MS for urinary metabolomics. While fecal microbiome alpha diversity showed no significant differences between groups, urinary metabolomics demonstrated distinct structural patterns and higher evenness in type 2 diabetes patients. The study identified several diabetes-associated urinary metabolites, including elevated levels of glucose and inositol, along with decreased levels of 6 urine metabolites including glycolic acid, hippurate, and 2-aminoethanol. In the fecal microbiome, genera such as *Escherichia*-*Shigella* showed positive correlation with type 2 diabetes, while *Lacticaseibacillus* demonstrated negative correlation. Receiver operating characteristic curve analyses revealed that urinary metabolites exhibited superior diagnostic potential compared to fecal microbiome features, with an area under the curve of 0.90 for the combined metabolite model versus 0.82 for the integrated bacterial taxa model. These findings suggest that urinary metabolomics may offer a more reliable approach for type 2 diabetes diagnosis compared to fecal 16S metabarcoding, while highlighting the potential of multi-marker panels for enhanced diagnostic accuracy.

## Introduction

Diabetes is a chronic metabolic disorder characterized by elevated blood glucose levels, leading to significant health complications and economic burdens worldwide [1, 2]. The diabetic population is expected to continue increasing; approximately 480 million individuals globally are affected by diabetes, with projections indicating a 25% increase by 2030 and a 51% surge by 2045 relative to current prevalence rates [3]. Type 2 diabetes is the most prevalent form of diabetes, accounting for approximately 90% of all diabetes cases, and primarily associated with insulin resistance and relative insulin deficiency [4, 5]. The health implications of type 2 diabetes are profound, encompassing increased risks of cardiovascular diseases, neuropathy, retinopathy, and nephropathy, which collectively contribute to reduced quality of life and heightened mortality rates [6-9]. The increasing prevalence of type 2 diabetes, coupled with its substantial socioeconomic burden, necessitates the development of more efficient diagnostic approaches and deeper understanding of its underlying pathophysiology.

Recent advances in high-throughput technologies have enabled comprehensive profiling of both the fecal microbiome and urine metabolome, offering new perspectives on their roles in type 2 diabetes pathogenesis. Urinary metabolomics has shown promise in identifying novel biomarkers for various metabolic disorders, including type 2 diabetes and diabetic kidney disease, offering a non-invasive approach to disease monitoring [10, 11]. Analogously, the gut microbiota has emerged as a crucial modulator of host metabolism, with mounting evidence suggesting its involvement in glucose homeostasis and insulin sensitivity [12-14]. While both 16S metabarcoding and urinary metabolomic analyses have been individually explored in relation to type 2 diabetes, systematic comparative studies evaluating their respective diagnostic potentials are limited.

In this study, we conducted a comparative analysis of fecal microbiome and urine metabolome profiles in type 2 diabetes patients (T2D group) and healthy controls (control group) to evaluate their respective discriminatory powers. This study focused specifically on fecal microbiome and urinary metabolome analyses, as these approaches offer complementary insights into host-microbe interactions and systemic metabolic alterations. The fecal microbiome analysis provides direct information about the microbial community structure potentially influencing glucose metabolism, while urinary metabolomics captures the downstream metabolic consequences of both host and microbial activities. This combination allows for a more comprehensive understanding of the metabolic dysregulation in type 2 diabetes compared to fecal metabolome or urinary microbiome analyses. By employing a multi-omics approach, we aimed to identify novel biomarkers and validate previously reported associations while assessing the potential of integrated marker panels for enhanced diagnostic accuracy. Furthermore, we investigated the structural characteristics of both fecal microbiome and urine metabolome to better understand their patterns in the context of type 2 diabetes, potentially revealing new insights into disease-associated biological alterations.

## Materials and Methods

### Study Cohort and Sample Collection

Initial study participants (*n* = 100) were recruited at Dangbom Korean Medicine Clinic between January 25 and February 29, 2024, after providing written informed consent. The cohort comprised adults aged 19-65 years, divided into type 2 diabetes and healthy control groups. T2D group inclusion criteria encompassed: medically diagnosed type 2 diabetes with more than 3 months of pharmaceutical treatment, absence of clinically significant findings, and voluntary consent. Control group criteria included: no diabetes history, absence of diseases based on medical screening, normal clinical findings, and voluntary consent. Exclusion criteria comprised: pregnant or nursing women, concurrent clinical trial participation, and investigator-determined unsuitability. Participants' fecal and urine samples were collected domestically, transported to the clinic, and stored at -80°C until analyses.

### Acquisition and Preprocess of Fecal Amplicon Aequencing Data

Fecal metagenomic DNA was isolated via the AccuFAST automated extraction system (AccuGene, Inc., Republic of Korea). The amplicon sequencing protocol targeted the V4 region of the 16S ribosomal RNA gene, employing 1.5 ng of the extracted fecal metagenomic DNA. The amplification was conducted in triplicate via 25 polymerase chain reaction cycles utilizing KAPA HiFi HotStart ReadyMix (Roche sequencing, USA), incorporating barcoded primers carrying Nextera adapter sequences (515fb/806rb), with appropriate negative controls. Subsequently, the amplified products underwent purification using HiAccuBead technology (AccuGene Inc.) following standardized protocols. Quantification and pooling of the prepared amplicons preceded sequencing, which was performed on the Illumina MiSeq platform using MiSeq Reagent Kit v2 (Illumina, USA) for 500 cycles, generating paired-end reads of 2 × 250 base pairs. The paired-end sequencing data, underwent quality control and trimming with the plugin q2-dada2 pipeline within QIIME2 [15, 16], facilitating the identification of distinct amplicon sequence variants (ASVs). The taxonomic classification of ASVs was performed using the classify-sklearn method in the plugin q2-feature-classifier with a pretrained Naïve Bayes classifier generated using the SILVA database v.138.1 [17]. Only classifications with a confidence score higher than 0.7 were retained to ensure taxonomic reliability.

### Acquisition of Urinal Metabolomic Data via GC–MS

100 ml of urine samples were mixed with 900 ml of methanol. After mixing thoroughly for 5 min, the samples were centrifuged at 13,000 rpm for 10 min at 4°C. 400 ml of the supernatant was then combined with 20 ml of a ribitol solution (internal standard, 0.5 mg/ml in water) and subjected to centrifugal vacuum concentration. To evaluate the stability and reliability of the analysis, quality control (QC) samples were prepared by blending 30 μl from each sample. The samples were then treated with 100 μl of methoxyamine hydrochloride (20 mg/ml in pyridine) and ultrasonicated using a Powersonic 520 (Hwashin, Republic of Korea) at 4°C for 20 min. Following a 1.5-min mixing period, the samples were incubated in the dark at 30°C with continuous shaking at 75 rpm for 90 min. The samples were subsequently silylated with 50 μl of N-methyl-N-(trimethylsilyl) trifluoroacetamide (MSTFA) and incubated with shaking at 75 rpm and 37°C for 30 min. Following the incubation, the samples were centrifuged at 12,000 rpm for 5 min at 4°C, and 80 μl of the supernatant was then transferred into individual vials for metabolite analysis. The derivatized samples were analyzed using a GC–MS-QP2020 (Shimadzu, Japan) with an RTX-5MS capillary column (Restek, USA). GC–MS conditions were as follows: injector temperature at 230°C; transfer line temperature at 250°C; helium flow rate of 1 ml/min, detector at 280°C. The GC oven temperature program began at 80°C, held for 2 min., then increased at 15°C/min to a final temperature of 330°C, held for 6 min. A 1 μl sample was analyzed in split mode (1:50) with an m/z range of 85–500 and electron impact ionization at 70 eV. To ensure stability, performance, and reproducibility, QC samples were analyzed alongside the test samples.

The retention index (RI) values of the compounds were calculated using the retention times of C7–C40 alkane standards (Sigma-Aldrich, USA) under identical GC–MS conditions. Raw peak data were processed and converted to ABF files using Shimadzu GC–MS Postrun Analysis (Shimadzu) and MS-DIAL v. 4.9. Identification was performed with a retention index tolerance of 20, an EI similarity cut-off of 90%, and an identification score cut-off of 90%, referencing EI-MS and Kovats RI. The identified spectra were further matched to the NIST v. 20.0 library and standard reagents. Peak intensities were normalized to ribitol.

### Statistical Analyses of the Omics Data

Statistical analyses and visualizations were performed in R 4.1.0. Diversity metrics were calculated using the vegan [18] and ape [19] packages with subsampled sequencing data considering the lowest read depth of the cohort samples. Permutational multivariate analysis of variance (PERMANOVA) was performed with adonis2 in the vegan package [18] based on Bray-Curtis and Jaccard indices. Taxonomic features specifically associated with diabetic or normal groups were identified using microbiome multivariable associations with linear models 2 (MaAsLin2) [20] and linear discriminant analysis effect size (LEfSe) [21] with a Kruskal-Wallis test alpha of 0.05 and a logarithmic linear discriminant analysis (LDA) score threshold of 2.5. To analyze the diagnostic accuracy of identified taxonomic or metabolomic features, receiver operating characteristic (ROC) curves were generated using the roc function in the pROC package [22] based on logistic regression models. Diagnostic performances of models were quantified and evaluated based on corresponding area under the curve (AUC). All analytic outcomes were visualized with ggplot2 package [23].

## Results and Discussion

### Compilation of Study Cohorts and Their Corresponding Omics Datasets

**Fig. 1:**
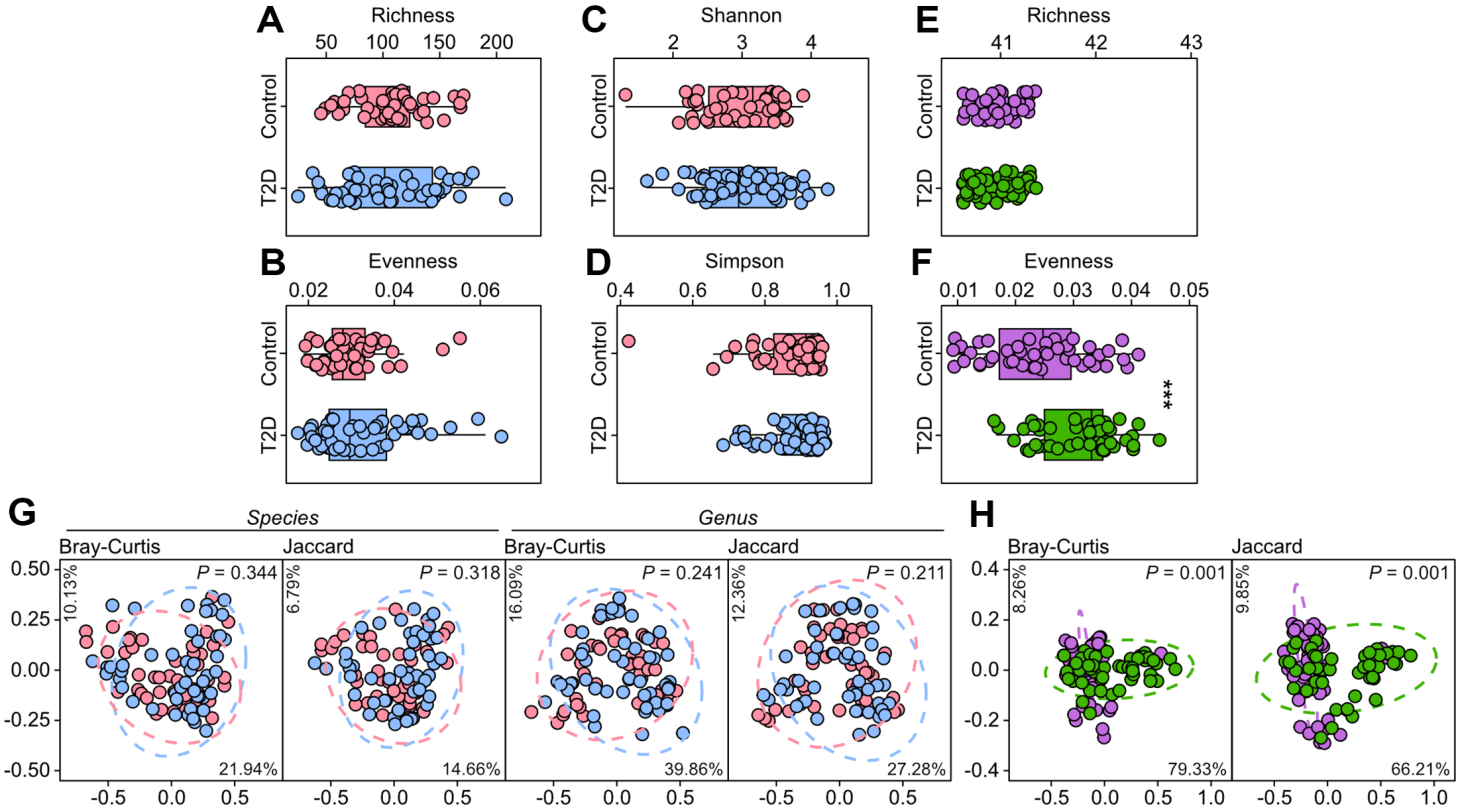
Structural comparison of the fecal microbiome and urine metabolome across subject groups. (**A**–**F**) Diversity levels of fecal microbiome (**A**–**D**) and urine metabolome (**E, F**) of the study cohort were computed and compared between the control and T2D groups. Statistical significances were indicated by asterisks (***p < 0.001, Wilcoxon rank sum test). (**G, H**) Principal coordinate analyses of the taxonomic structures of the fecal microbiome at species and genus levels determined by 16S rRNA amplicon sequencing (**G**), and the urine metabolome (**H**) of the control and T2D groups, based on Bray-Curtis and Jaccard indices. P values of comparisons between the groups computed via permutational analysis of variance.

The initial cohort comprised 100 participants, which included a 10% overcollection to account for potential dropouts; the baseline sample size (*n* = 82) was calculated using G*Power, with parameters set at: correlation *p_H1_* = 0.3, α = 0.05, power = 0.80, tested predictors = 2. Participants were recruited to maintain a 1:1 ratio between the diabetes patient group and the normal control group. From the initial cohort of 100 participants, 4 withdrew consent and 2 were excluded due to sample quality issues, resulting in a final cohort of 94 subjects comprising 46 in the control group and 48 in the T2D group (Table S1). Age distribution and sex ratio of the T2D group and control group were described in Table S1. fecal microbiome analysis was conducted via 16S rRNA amplicon sequencing, and demultiplexed sequence depths ranged from 45,295 to 384,091 reads (median 113,196 reads). Sufficient sequencing depth for the diversity analyses based on 16S metabarcoding (Fig. 1A–1D) was confirmed through rarefaction curves based on alpha diversity indices (Fig. S1).

### Potency Comparison of Fecal Microbiome and Urine Metabolome Structures in Distinguishing Diabetics from Healthy Individuals

We first investigated structural characteristics of fecal microbiome, together with those of urine metabolome, to assess their potential for differentiating individuals with diabetes from healthy controls. Comparison of fecal microbiome alpha diversity levels did not show noticeable distinction between the two groups (Fig. 1B–1E). In the case of urine metabolome, the two groups showed similar levels of richness, namely the number of total detected metabolites (Fig. 1F). However, evenness was significantly higher in diabetics compared to healthy individuals (Fig. 1G), suggesting more even distribution of detected metabolite abundances in type 2 diabetics. This increased evenness may indicate a more balanced metabolic profile in T2D patients, where less metabolites predominate. Such findings could suggest a shift in metabolic pathways in type 2 diabetes, which may involve altered metabolic regulation and homeostasis. Previous metabolomics studies have indicated that individuals with type 2 diabetes exhibit distinct urinary metabolite profiles compared to healthy individuals, but these differences do not necessarily correspond to higher alpha diversity in urine metabolites [11, 24]. Unlike microbiome studies, alpha diversity is not a commonly used analytical metric in metabolome analyses, and thus far, there have been no reported cases linking the evenness of urinary metabolomic structure to type 2 diabetes.

**Fig. 2:**
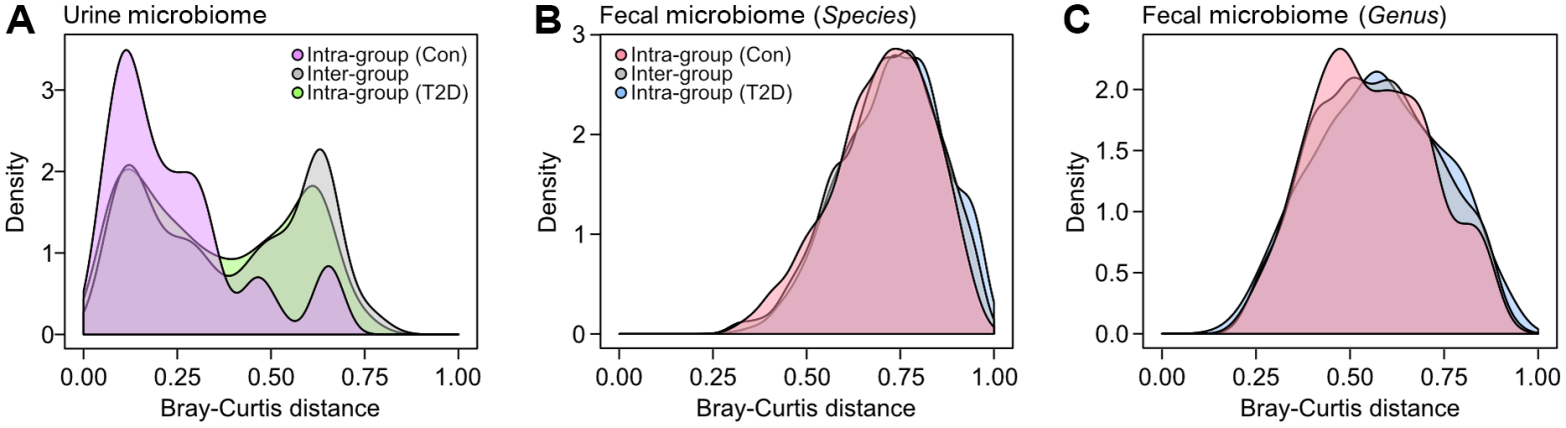
Distribution of inter- and intra-group dissimilarities in the urine metabolome and fecal microbiome. (**A**) Inter- and intra-group Bray-Curtis dissimilarities of the urine metabolomic structures were computed and visualized as density plots. (**B, C**) Same analyses were performed with fecal microbiome at species (**B**) and genus (**C**) levels.

We additionally assessed the structural distinctiveness between diabetics and healthy individuals regarding both fecal microbiome and urine metabolome, via principal coordinates analysis (PCoA) with both Bray-Curtis and Jaccard indices. Considering the low taxonomic resolution of 16S rRNA amplicon sequencing at the species level [25-27], not only species but genus level data also were employed. Similar to the above-mentioned alpha diversity analyses, diabetics and healthy individuals were not statistically discernable (PERMANOVA, *p* < 0.05) in all analyses based on fecal microbiome architectures at both species and genus levels (Fig. 1H). On the other hand, the two groups showed noticeable urine metabolomic separation (Fig. 1I), corroborating a previous demonstration about the capability of urine metabolomics as a valuable approach for the diagnosis of type 2 diabetes [28-30]. We further looked into the structural differences in fecal microbiome and urine metabolome between the control and T2D groups by computing intra- and inter-group structural dissimilarities. In the case of urine metabolome, healthy individuals in the control group exhibited relatively low intra-group dissimilarity distribution than that of the T2D group (Fig. 2A), suggesting more structural similarity within the control group. Both intra-group distribution of the T2D group and inter-group distribution (control group vs. T2D group) showed bimodal distribution patterns, reflecting a polarized structural pattern within the T2D group (Figs. 1H and 2A). Overall, the intra-group dissimilarity of urine metabolomic structures within the control group was statistically distinctive from other two distributions (Kruskal-Wallis test with Benjamini-Hochberg FDR correction, FDR < 0.001). However, the intra- and inter-group distributions of structural dissimilarities of fecal microbiome 16S metabarcoding were more comparable than those of the urine metabolomic structures at both species and genus levels (Fig. 2B and 2C).

Nevertheless, the sex-specific characteristics of the urinary metabolome cannot be disregarded, as they arise from fundamental differences in physiology, hormonal regulation, and metabolic capacity between males and females [31, 32]. Given the statistically significant difference in sex distribution between the T2D and control groups (Table S1, *P* = 0.0126, Chi-squared test), PCoA of the urinary metabolome was stratified by sex. The analysis revealed statistically significant differentiation in urinary metabolomic profiles between males and females, whereas age-based stratification showed no significant separation, suggesting that sex represents a critical confounding variable (Fig. S2).

### Metabolite Biomarkers and Their Correlations with Type 2 Diabetes Revealed by Urinary Metabolomic Profiling

**Fig. 3:**
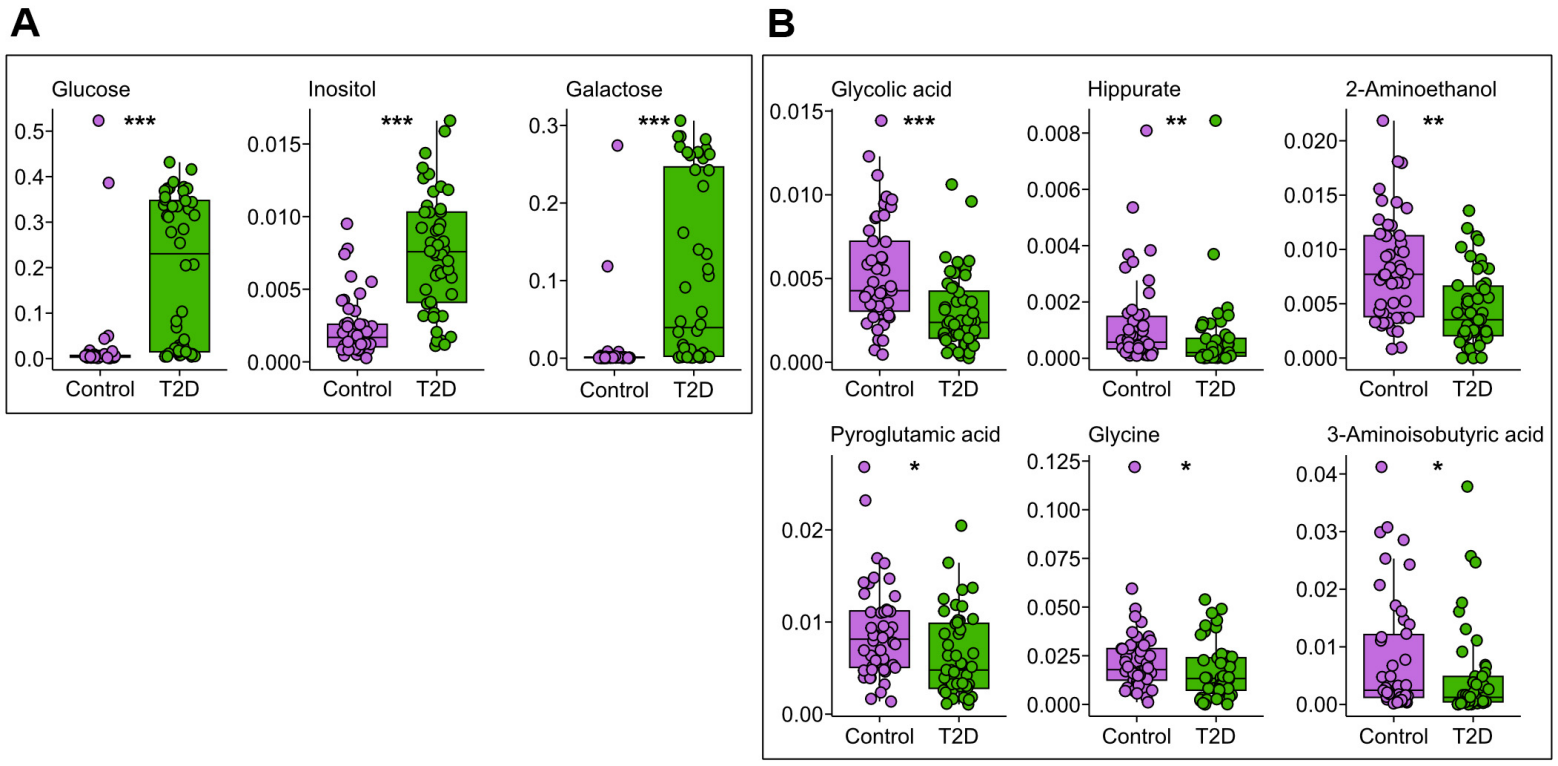
Identification of urine metabolites distinguishing individuals with type 2 diabetes from healthy individuals. (**A**) 3 urinary metabolites were identified as markers positively associated with type 2 diabetes. (**B**) 6 urinary metabolites were screened as markers negatively correlated with type 2 diabetes. Metabolite identification was performed using multivariable association with linear models (MaAsLin2, FDR < 0.05). Wilcoxon rank sum tests were performed for the group comparison (*p < 0.05, **p < 0.01, ***p < 0.001).

Given the observed structural distinctions in urinary metabolomic profiles between diabetic and control groups, we aimed to detect urinary metabolites specifically correlated with type 2 diabetes using generalized linear models via MaAsLin2 [20]. Both age and sex were implemented as random effects in the analyses. The analysis determined glucose, inositol, and galactose as the urine metabolites positively correlated with type 2 diabetes (Fig. 3A). It also determined multiple urine metabolites which were negatively correlated with type 2 diabetes, such as glycolic acid, hippurate, 2-aminoethanol, pyroglutamic acid, glycine, and 3-aminoisobutyric acid (Fig. 3B). The measured intensity levels of selected urinary metabolites were statistically verified for differences between the diabetes and healthy groups (Fig. 3). Among the identified urinary metabolites, while some were previously established as well-known biomarkers for type 2 diabetes, others had not demonstrated clear associations with type 2 diabetes in prior research.

Glucose in urine, namely glucosuria, has been known as a definite diabetes-specific symptom resulting from hyperglycemia [33, 34]. Similarly, in the case of inositol, myo-inositol, the most abundant inositol isomer, was also reported as a urinary metabolite elevated in diabetics due to the insulin resistance [35, 36]. Interestingly, galactose levels in urine were significantly discriminated between the diabetes and control groups in our cohort, although direct evidence for urinary galactose as a type 2 diabetes marker has not been reported. The lower level of glycolic acid in urine has been considered as an indicator of diabetic kidney disease, such as glomerular lesions [37-39]. Reduced urinary excretion of pyroglutamic acid, also known as 5-oxoproline or polyglutamic acid, serves as a significant biomarker in the pathophysiology of chronic kidney disease, a renal dysfunction frequently caused by type 2 diabetes [39-41]. In addition, reduced urinary excretion of pyroglutamic acid may indicate glycine insufficiency [42]. Decreased plasma glycine level has been considered as a biomarker for reduced glucose tolerance and type 2 diabetes [43, 44], but the correlation between urinary glycine and type 2 diabetes has not been uncovered yet. On the other hand, previous metabolomic investigations have demonstrated reduced levels of urinary hippurate, a glycine conjugate of benzoic acid, in individuals with diabetes and glucose tolerance impairment compared to healthy individuals controls [43, 45, 46]. While urinary levels of 2-aminoethanol, also known as ethanolamine, demonstrated significant differentiation between the study groups, no direct association with type 2 diabetes has been established to date. Nevertheless, urinary 2-aminoethanol has been identified as a potential biomarker for diabetic retinopathy [47, 48]. Similarly, 3-aminoisobutyric acid showed noticeable distinction between urines of the two groups, but no previous study demonstrated its lower urinary level as a biomarker of type 2 diabetes. Still, 3-aminoisobutyric acid has demonstrated beneficial effects on glucose metabolism and insulin sensitivity in type 2 diabetic mice, although the underlying molecular mechanisms mediating these metabolic improvements remain to be fully elucidated [49, 50].

### Profiling of Type 2 Diabetes-Specific Fecal Microbiome Taxa: Identification of Novel Taxonomic Features and Validation of Established Markers

**Fig. 4:**
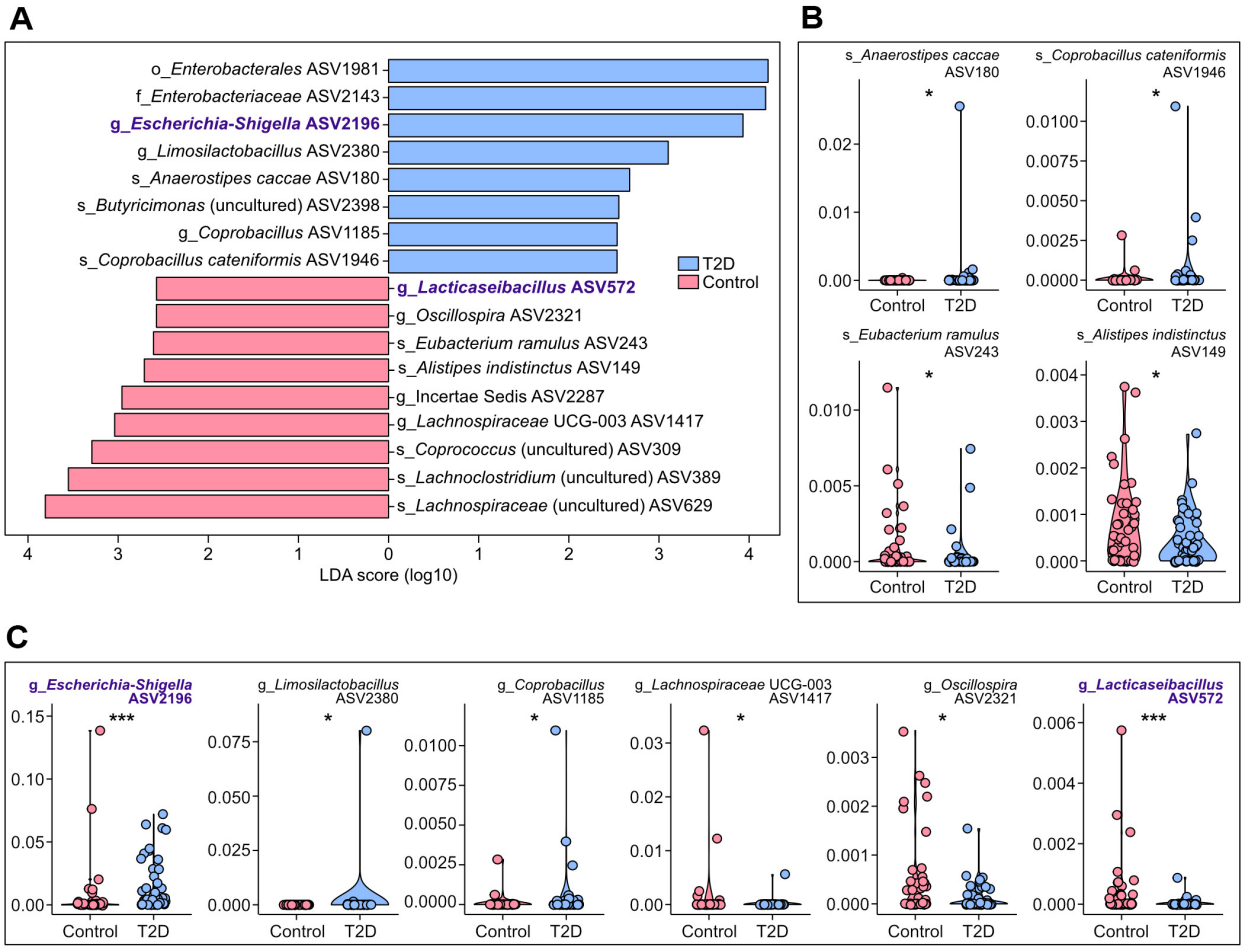
Taxonomic features of fecal microbiome distinguishing individuals with type 2 diabetes from healthy individuals identified via Linear discriminant analysis effect size (LEfSe). (**A**) Screened taxonomic features identified by LEfSe from fecal microbiome 16S rRNA amplicon sequencing data with all taxonomic hierarchy levels. (**B, C**) Comparisons of relative abundances of identified species features (**B**) and genus features (**C**). Wilcoxon rank sum tests were performed for the group comparison (*p < 0.05, **p < 0.01, ***p < 0.001). The taxonomic features, which were screened by MaAsLin2 as well (FDR < 0.05), were highlighted in purple. Raw ASV IDs and confidence information of the selected features were summarized in Table S2.

In line with the lack of statistical significance on structural differences between fecal microbiome of the two groups, the general linear model-based analysis with age and sex as random effects identified only 2 genera exhibiting significant associations with diabetes status. Specifically, the ASV corresponding to *Escherichia*-*Shigella* demonstrated a positive correlation with diabetes (FDR = 0.00174), while another ASV assigned to *Lacticaseibacillus* showed a significant negative correlation (FDR = 0.0304). We repeated the screening through another statistical approach utilizing LDA, namely LEfSe, including all taxonomic hierarchy levels. The new analysis identified multiple taxonomic features specific to diabetic or control groups in varied taxonomic hierarchy levels (Fig. 4A). We excluded selected taxonomic features positioned above the genus level in the taxonomic hierarchy or had ambiguous taxonomic information were excluded from further analyses due to their limited utility as biomarkers.

At the species level, ASVs classified as *Anaerostipes caccae* and *Coprobacillus catenaformis* were identified as positively diabetes-associated species, while ASVs corresponding to *Eubacterium ramulus* and *Alistipes indistinctus* were identified as negatively diabetes-associated species (Fig. 4B). Intriguingly, all these 4 species have been characterized as butyrate-producing bacteria [14, 51-54]. As butyrate production in the gut exerts significant effects on gut integrity and metabolic regulation, changes in relative abundances of these bacteria may be linked to metabolic pathophysiology. Indeed, multiple investigations of the gut microbiome have revealed a diminished abundances of butyrate-producing bacteria in subjects with type 2 diabetes [14, 55, 56]. However, this metabolic association is not universally observed across all bacteria capable of butyrate production in the gut, despite the predominant pattern of metabolically advantageous characters among analyzed butyrate-producing taxa [14]. In this context, the observed positive association between diabetic status and relative abundances of *A. caccae* and *C. catenaformis* suggests these species may possess metabolic functionalities extending beyond butyrate synthesis, potentially encompassing alternative metabolic cascades or microbiome-mediated homeostatic regulation. Elucidation of their comprehensive metabolic repertoire and mechanistic implications in type 2 diabetes pathophysiology warrants further investigation.

At the genus level, the analysis via LEfSe revealed positive correlations between diabetes and three ASVs: *Escherichia*-*Shigella*, *Limosilactobacillus*, and *Coprobacillus* (Fig. 4C). Notably, *Escherichia*-*Shigella* emerged as a diabetes-specific taxonomic biomarker again. This finding aligns with previous study demonstrating that individuals with metabolic disorders, including diabetes, typically exhibit increased fecal microbiome instability, often characterized by elevated levels of facultative pathogens such as *Escherichia*-*Shigella* [57]. While a number of *Limosilactobacillus* strains have been documented to facilitate glucose homeostasis under diabetic conditions [58-60], the elevated abundance of this genus in diabetic subjects was observed in our study (Fig. 4C). Intriguingly, our observation of increased relative abundance of *Coprobacillus* in diabetic subjects (Fig. 4C) contrasted with previous research that reported a negative correlation between this genus and diabetes status [61]. In the meantime, three genus level ASVs demonstrated negative correlations with diabetes, such as ASVs assigned to *Lachnospiraceae* UCG-003, *Oscillospira*, and *Lacticaseibacillus* (Fig. 4C), which was identified as a genus marker negatively associated with type 2 diabetes via MaAsLin2. While *Lachnospiraceae* UCG-003 has not previously been linked to type 2 diabetes, the inverse relationship between *Oscillospira* and type 2 diabetes corroborates the earlier finding [62]. Although *Lacticaseibacillus* strains have demonstrated antidiabetic properties, potentially contributing to type 2 diabetes management through microbiome regulation, glucose metabolism improvement, and inflammation reduction [63], their negative correlation with type 2 diabetes had not been empirically validated prior to this investigation.

### Comparative ROC Analyses of Multiomic Markers for Type 2 Diabetes Classification

**Fig. 5:**
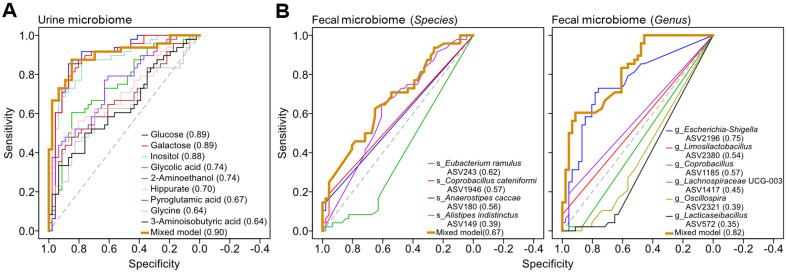
Receiver operating characteristic (ROC) curve analyses of the identified urine metabolome and fecal microbiome features for type 2 diabetes prediction. ROC curves of selected urine metabolomic features (**A**) and fecal microbiome taxonomic features determined via 16S metabarcoding (**B**; left, species level; right, genus level) in distinguishing between the control and T2D groups. Each curve represents the diagnostic performance of individual biomarkers or multivariable prediction models. The area under the curve (**AUC**) values and optimal cutoffs reflect the ability of the urine metabolome and fecal microbiome to discriminate between groups. The AUC values of the ROC curves for each feature are indicated in parentheses following the respective feature names. The dashed line indicates the random classification boundary (AUC = 0.5).

We performed ROC curve analyses to assess the discriminatory capacity of the key urine metabolomic and fecal microbiome taxonomic features between control and diabetic cohorts. We first evaluated the diagnostic utility of the selected urinary metabolites (Fig. 5A). Overall, the selected metabolites demonstrated robust predictive values, which were quantitated via AUC. Especially, metabolites correlated positively with type 2 diabetes, namely glucose, galactose, and inositol (Fig. 3A), demonstrated considerable performance as diagnostic markers compared to other metabolites showed negative correlations with diabetes (Fig. 5A). To assess the collective diagnostic capacity of the identified urinary metabolite features, a logistic regression model was constructed, employing selected urinary metabolite features as predictors and type 2 diabetes status as the dependent variable. The resulting multivariable prediction model’s ROC curve revealed that the consolidated diagnostic performance demonstrated slightly enhanced diagnostic accuracy relative to individual metabolites that exhibited positive correlation with type 2 diabetes (Fig. 5A, AUC = 0.90). ROC curve analyses of discrete fecal microbial taxa demonstrated differential predictive capabilities between species and genus levels. The comparatively lower diagnostic accuracy observed in the species feature-based model further corroborates the inherent limitations of 16S rRNA amplicon sequencing regarding species-level resolution [25-27]. Of particular significance, the ASV assigned to *Escherichia*-*Shigella*, one of diabetic-specific genera identified by both MaAsLin2 and LEfSe, demonstrated considerable discriminatory power with AUC values exceeding 0.75. The implementation of an integrated multi-taxa model demonstrated noticeably enhanced diagnostic accuracy compared to individual taxonomic markers in discriminating between diabetic and control groups (AUC = 0.82). Our analyses revealed the better diagnostic potential of urinary metabolites compared to fecal microbiome features in discriminating type 2 diabetes status. Notably, while individual metabolites like glucose, galactose, and inositol showed strong predictive performance, the integration of multiple biomarkers—whether metabolites or bacterial genera—consistently yielded enhanced diagnostic accuracy, suggesting the value of a multi-marker approach for robust disease classification [64].

## Conclusion

The current study demonstrated the better discriminatory power of urinary metabolomics and potential of fecal microbiome profiles in distinguishing type 2 diabetes. While our analyses identified both novel and previously reported biomarkers in both urine metabolome and fecal microbiome, the consolidated multi-marker approach showed enhanced diagnostic accuracy compared to individual markers. The identification of novel type 2 diabetes-associated metabolites and bacterial taxa opens new avenues for investigating their mechanistic roles in diabetes pathophysiology. Still, the sample size and geographical concentration of subjects in this study may limit the generalizability of our results to broader populations. Future research should focus on validating these markers in larger, diverse cohorts and exploring their potential integration into clinical diagnostic platforms. Additionally, longitudinal studies investigating the temporal dynamics of these biomarkers during diabetic progression could provide valuable insights for early detection and preventive interventions against type 2 diabetes.

## Supplemental Materials

Supplementary data for this paper are available on-line only at http://jmb.or.kr.
