# Supplementary material for: Fecal Microbiome and Urine Metabolome Profiling of Type 2 Diabetes
Source: J Microbiol Biotechnol. 2025 Mar 11;35:e2411071. doi: 10.4014/jmb.2411.11071 (PMC11985407; doi:10.4014/jmb.2411.11071)
Supplement: Supplementary file 1 [file jmb-35-e2411071-supple.pdf]

<u>Supplementary Information</u> for:

"Fecal microbiome and urine metabolome profiling of type 2 diabetes"

Hye-Min Yi[1,2†], Seok Won[3†], Juhan Pak[4], Seong-Eun Park[4], Mi-Ri Kim[2], Ji-Hyun Kim[2], Eun-Young Park[2], Sun-Young Hwang[1], Mee-Hyun Lee[1*], Hong-Seok Son[4*], and Suryang Kwak[3*]

[1]College of Korean Medicine, Dongshin University, Naju 58245, Republic of Korea
[2]Dangbom Korean Medicine Clinic, Seoul 03192, Republic of Korea
[3]Department of Bio and Fermentation Convergence Technology, College of Science and Technology, Kookmin University, Seoul 02707, Republic of Korea
[4]Department of Biotechnology, College of Life Sciences and Biotechnology, Korea University, Seoul 02841, Republic of Korea

† These authors contributed equally to this work.

* Corresponding authors
Mee-Hyun Lee
(Phone: +82-61-330-3516, Fax: +82-61-330-3519, E-mail: mhlee@dsu.ac.kr)
Hong-Seok Son
(Phone: +82-2-3290-3053, Fax: +82-2-3290-3040, E-mail: sonhs@korea.ac.kr)
Suryang Kwak
(Phone: +82-2-910-5735, Fax: +82-2-910-5739, E-mail: skwak@kookmin.ac.kr)

This PDF file includes:
2 Supplementary figures
2 Supplementary tables

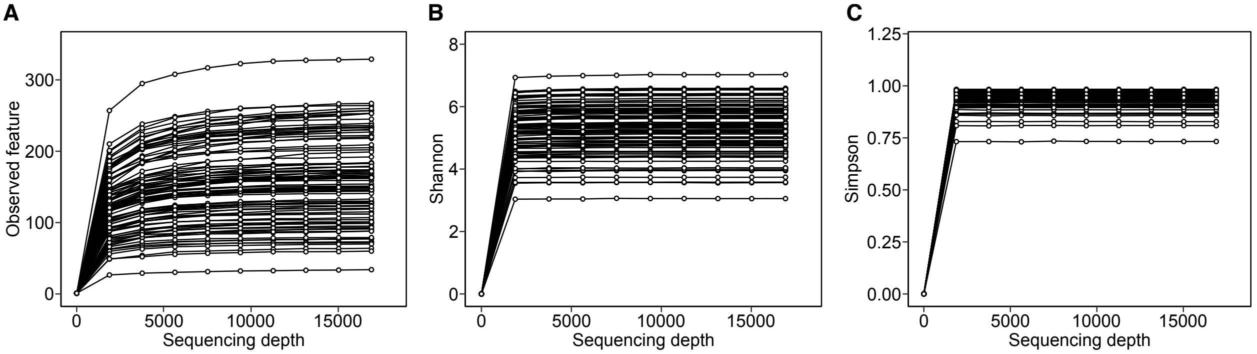

Figure S1. Rarefaction analysis of sequencing data of this study based on alpha diversity indices. The number of observed features (richness, A), Shannon (B), and Simpson indices (C) were calculated after taxonomic classification via 16S metabarcoding. All analyses reached plateau before the depth level of 16,900 reads.

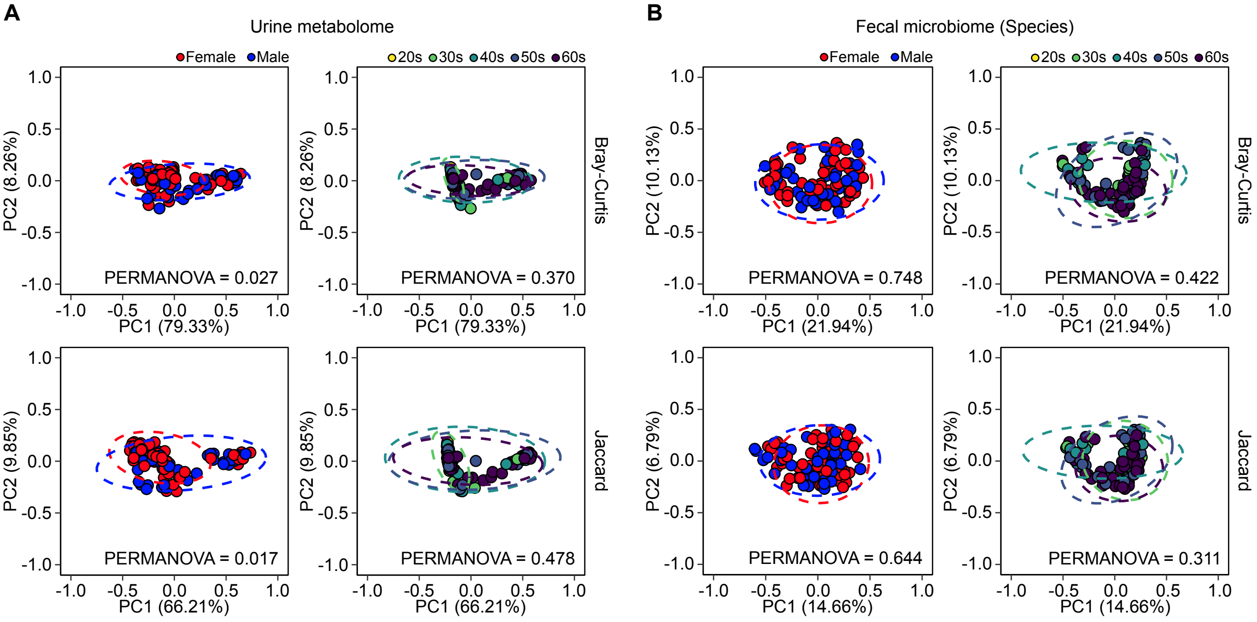

Figure S2. Structural comparisons of urine metabolome and fecal microbiome based on metadata variables. Principal coordinate analyses of fecal microbiome taxonomic compositions derived from 16S rRNA amplicon sequencing (A) and urinary metabolomic profiles (B), stratified by sex (left) and age (right). Analyses were performed using both Bray-Curtis (upper panel) and Jaccard (lower panel) dissimilarity metrics.

Table S1. Age distribution and sex ratio of the T2D group and control group

	T2D (n = 48)	Normal (n = 46)
Age: Mean (±SD, range)	58.31 (±6.51, 36-66)	49.13 (±11.46, 29-66)
Sex (female%)	39.58	67.39

Table S2. The mapping table of raw ASV IDs, labels in the main text, and confidence of selected taxonomic features (related Figs 4 and 5).

ASV ID	Label	Confidence
c77d35bc5d04d691cc4ffa9cf9b26d2e	ASV1981	0.925967114
d22ff5b00dd694dd58062583920fcda0	ASV2143	0.954416292
918bdabd144898af1dfe73c34ad6e752	ASV2196	0.912257796
3bb5a29c00279639524f84d649440e56	ASV2380	0.973825278
fec6f192e64feb5c8c18976bcdb6e44e	ASV180	0.731064387
720f9b1c2eebe5ea9cd3a1ee80635931	ASV2398	0.995712278
6405966f04d67b906f89d2292f33283c	ASV1185	0.999984712
cf50543ac0434eac09b80813ebdf1667	ASV1946	0.705142153
be98d8ff969d6a63f02a1689d8b0ba87	ASV572	0.999992276
e6af6f0383ee7e8e93b2b6e9efbaf68d	ASV2321	0.996411423
3a996e2f3bd01dc272a98a12ff8d383d	ASV243	0.702719942
9037a8807f265f6926427153c9f6f6f8	ASV149	0.994923584
6f257e816893b97fdd9284fbaa9dce1e	ASV2287	0.99999855
fa3b030e4b17bb754f30690a710dfee7	ASV1417	0.974841873
ed7747f5b0284274c95a92cc42e8657d	ASV309	0.84302599
cbe2abdf95ba4f41c1453328d1e0230c	ASV389	0.851888796
51ef6c3bec7f255e9748a69e884c9c9f	ASV629	0.990983256
